# Circular and Micro RNAs from *Arabidopsis thaliana* Flowers Are Simultaneously Isolated from AGO-IP Libraries

**DOI:** 10.3390/plants8090302

**Published:** 2019-08-26

**Authors:** Érika Frydrych Capelari, Guilherme Cordenonsi da Fonseca, Frank Guzman, Rogerio Margis

**Affiliations:** 1 Programa de Pós-graduação em Biologia Celular e Molecular (PPGBCM), Centro de Biotecnologia, Universidade Federal do Rio Grande do Sul, Porto Alegre 91501-970, Brazil; 2 Programa de Pós-graduação em Genética e Biologia Molecular (PPGBM), Universidade Federal do Rio Grande do Sul, Porto Alegre 91501-970, Brazil; 3 Centro de Biotecnologia, Laboratório de Genomas e Populações de Plantas (LGPP), Universidade Federal do Rio Grande do Sul, Av. Bento Gonçalves, 9500—Laboratório 206 Prédio 43422, Porto Alegre 91501-970, Brazil

**Keywords:** circRNA, microRNA, non-coding RNA, argonaute, immunoprecipitation, plant

## Abstract

Competing endogenous RNAs (ceRNAs) are natural transcripts that can act as endogenous sponges of microRNAs (miRNAs), modulating miRNA action upon target mRNAs. Circular RNAs (circRNAs) are one among the various classes of ceRNAs. They are produced from a process called back-splicing and have been identified in many eukaryotes. In plants, their effective action as a miRNA sponge was not yet demonstrated. To address this question, public mRNAseq data from Argonaute-immunoprecipitation libraries (AGO-IP) of *Arabidopsis thaliana* flowers were used in association with a bioinformatics comparative multi-method to identify putative circular RNAs. A total of 27,812 circRNAs, with at least two reads at the back-splicing junction, were identified. Further analyses were used to select those circRNAs with potential miRNAs binding sites. As AGO forms a ternary complex with miRNA and target mRNA, targets count in AGO-IP and input libraries were compared, demonstrating that mRNA targets of these miRNAs are enriched in AGO-IP libraries. Through this work, five circRNAs that may function as miRNA sponges were identified and one of them were validated by PCR and sequencing. Our findings indicate that this post-transcriptional regulation can also occur in plants.

## 1. Introduction

The advancements in high throughput sequencing technologies and the development of new bioinformatics tools expanded the knowledge about non-coding RNAs (ncRNAs) and their functions as regulators of gene expression. The ncRNAs can be subdivided into two major classes: (i) small non-coding RNAs (sncRNAs) and (ii) long non-coding RNAs (lncRNAs) [1]. The lncRNAs are usually more than 300 nucleotides in length and can be regulated by microRNAs (miRNAs) [2,3]. miRNAs represent small RNAs, with approximately 19–24 nucleotides, and their main function is to act as a post-transcriptional gene regulator, through the RNA Induced Silencing Complex (RISC). Argonaute (AGO) is the main protein involved in this regulatory complex. It harbors small RNAs in its active site and promotes the interaction between the miRNA sequence and the target messenger RNA (mRNA), forming a ternary miRNA:AGO:mRNA complex. It leads to a repression in gene expression either by the mRNA cleavage or by translational repression [4]. The regulation mediated by miRNA occurs through the base pairing of complementary sequences, known as miRNA response elements (MREs), between mRNA and miRNA [5].

Competing endogenous RNAs (ceRNAs) are transcripts of coding or non-coding genes that have MREs and can compete with mRNA targets for miRNAs binding. They can promote a reduction in miRNA action by decreasing their availability in the cytoplasm [6,7,8,9]. A typical example of this mechanism is represented by ncRNA IPS1, which interacts with miR-399 by mimicking the MRE of its target mRNA PHO2, in a mechanism called target mimicry [10]. It has been suggested that an interaction network exists among ceRNAs, which communicate and co-regulate themselves through competition for a limited set of miRNA [7]. Therefore, all transcripts that share similar MREs can potentially compete for a specific miRNA.

A distinct class of newly discovered endogenous non-coding RNA was denominated as circular RNA (circRNA) [11,12]. In the early 1990s, due to their low levels of expression, circRNAs were considered as being splicing artifacts, corresponding to transcripts with scrambled exon order and splicing errors [13,14,15]. circRNAs were also associated with pathologic agents like hepatitis delta virus (HDV) [16] and plant viroids [17]. With the advent of next-generation sequencing technology and bioinformatic tools, the identification, biogenesis, and functions of circRNAs have been described, allowing a better understanding of these molecules [18,19]. Thus, many circRNAs were shown to be expressed as abundant and stable molecules [18] in different organisms, like humans [20,21], animals [12,22], yeast [23], bacteria [24], and plants [19,23,25,26,27,28,29]. In addition, circRNAs exhibited development-specific, tissue-specific and cell type-specific expression in animals, suggesting a regulatory role [18,30,31].

CircRNAs are characterized by the lack of 5′ caps and 3′ poly-A tails. Instead, they form a covalently closed loop structure originated by back-splicing circularization in a mechanism mediated by the spliceosomes. In this process, the 3’ region of a downstream exon of a given gene is linked to the 5’ region of an upstream exon of that same gene. The circularization enhances the RNA stability, making circRNAs resistant to RNase R, an exonuclease that degrades linear RNAs [32]. Due to this stability, some exonic circRNAs have been shown to be at higher concentrations than their linear counterparts [18,33,34]. circRNAs can be originated from exons [15,34], introns [12,35] or both [36]. However, most of the circRNAs are originated from exons of protein-coding genes [37]. Thus, circRNAs may comprise a single or multiples exons.

Another feature of circRNAs that has aroused great interest is its multi-functionality. circRNAs have been implicated in: (i) regulation of RNA processing [22,38], (ii) transcription regulation [39], (iii) interaction with RNA binding proteins and ribonucleoproteins complexes [40,41], and (iv) acting as microRNA sponges, preventing miRNAs to bind their target mRNAs [12]. Furthermore, miRNA binding sites in circRNAs are less likely to have polymorphisms than flanking sequences or random sites, suggesting an important role of circRNAs in the regulation of miRNA activities [42]. Up to now, the study of circRNAs in plants has received much less attention, compared to the wide comprehensive knowledge of circRNAs in mammals, in which a large number of circRNAs have been identified and characterized [43].

Recent studies have shown that circRNA are present in many species of plants [19,23,25,26,27,29]. However, it was not yet demonstrated whether they could effectively act as miRNA sponges. To address this question, we used a publicly available sequencing data from an Argonaute-immunoprecipitation experiment (AGO-IP) from *Arabidopsis thaliana* flowers followed by sequencing of the associated RNAs [44] to screen for circRNAs with miRNA binding sites. In the present work, five putative circRNAs that may function as miRNA sponges were found, with one of them being validated by PCR and sequencing. Our findings suggest the existence of AGO-miRNA-circRNAs complexes, and contribute another step in the understanding of post-transcriptional regulation mechanisms in plants.

## 2. Results

### 2.1. Identification of circRNAs in AGO-IP Libraries

**Figure 1:**
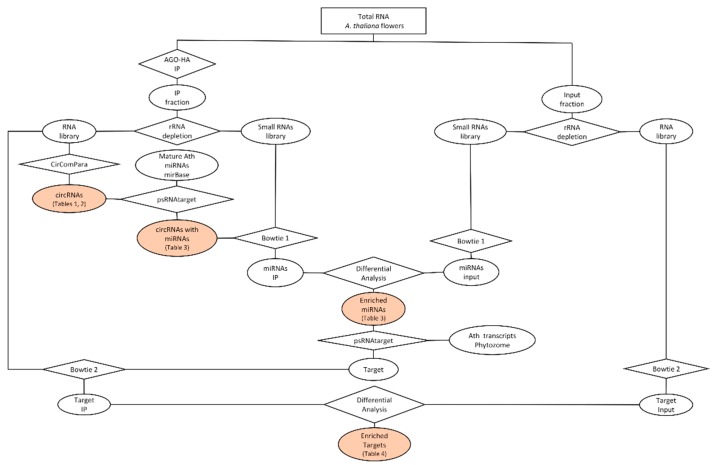
Flowchart for identification of circRNAs, miRNAs and target mRNAs in AGO-IP and control libraries. The total RNA from *A. thaliana* flowers was divided in two fractions. One of them went through Argonaute immunoprecipitation (IP fraction) and the other was used as control (Input fraction). Different methodologies are represented by rhombus, while the outputs are represented by ellipses. Filled ellipses correspond to results also presented in tables.

Circular RNAs with potential to act as sponges for miRNAs were identified in RNAseq data from libraries prepared from total RNA extracted from flowers A. thaliana. In a previous study, Carbonel and coworkers produced three independent libraries corresponding to Argonaute immunoprecipitation (AGO-IP) libraries [44]. These libraries were used in our analyses. Two lines of A. thaliana overexpressing the Argonaute wild type (DDH) and another overexpressing a mutant line with no ability to slice (DAH). Specific AGO-IP was carried using monoclonal antibodies directed against the human influenza hemagglutinin (HA) sequence tag present in the recombinant AGO (Figure 1).

The use of CirComPara allowed the identification of up to 29.358 circRNAs in AGO-IP RNAseq libraries (Table 1). Using the CircExplorer2 with the Segemehl anchor 86 putative circRNAs were identified, while using the Star anchor 15 and with TopHat, 23. The number of predicted circRNAs identified by FindCirc algorithm was 1422 and by the TestRealign was 27.812. The number of circRNAs hits is reduced to only three when certain methods that are more stringent are used (Table 1).

So far, we decided to focus on those circRNAs identified by at least three different methods. The description of these 12 circRNAs, including the library from which they were identified, the locus and function of parental gene, their origin and length are described in Table 2. The coordinates of the 12 circRNAs in the A. thaliana genome is listed in Supplementary Table S3. The majority of circRNAs was originated from perfect exon back-splicing, while two were produced from introns and another resulted from an imperfect exon back-splicing. The number of exons that form the chosen circRNAs varied from one to four. Their sizes ranged from 49 nt (At5g16880) to 1063 nt (At2g42170).

### 2.2. circRNAs with miRNA Binding Sites

In order to identify those plants circRNAs that can function as miRNA sponges, only the five circRNAs that have binding sites to miRNAs were selected, among the 12 previous circRNAs (Table 3). The read count of each of the 12 circRNA, matching the back-splicing junction, was analyzed in both AGO-IP and control libraries, in order to identify the enrichment in the AGO-IP (Table 3). In total, AGO-IP libraries had 284,490,887 reads, while the control library had 594,458,195. From the 428 mature A. thaliana miRNAs, 14 miRNAs were predicted as having at least one of the five circRNAs as targets. 10 from these miRNAs were more abundant in AGO-IP libraries (highlighted with an *) in comparison to input library (Table 3). All the miRNAs that have predicted sites of translational inhibition were enriched. Those with cleavage sites were poorly represented or not detected at any library.

### 2.3. The circRNAs Harbor Reverse Complementary Sequences of miRNAs which Targeted mRNAs Present in AGO-IP Libraries

The enriched miRNAs were selected to evaluate if their predicted target mRNAs were also present and enriched in AGO-IP libraries. In total, 260 mRNA targets were identified with an expectation range from 0.5 to 3 (Supplementary Table S2). Six out of the 10 enriched miRNAs presented mRNA targets with reads that were significantly more frequent in AGO-IP libraries than in the control input, reducing the number of predicted targets to 64 (Table 4).

### 2.4. circRNAs Validation by RT-PCR and Sequencing

**Figure 2:**
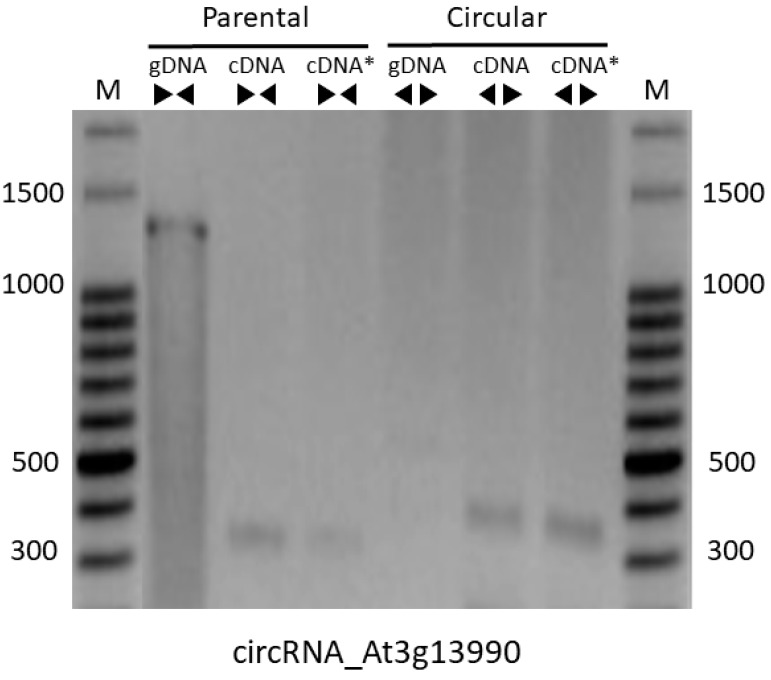
Validation of circRNA by RT-PCR. PCR reactions were performed using divergent primers (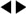) to amplify the circRNA_At3g13990. Convergent primers (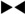) were used to amplify parental mRNA. Genomic DNA (gDNA) was used as control. Samples were analyzed on 1,5% agarose gel. (M) DNA size marker of 100 bp; cDNA: complementary DNA; cDNA*: complementary DNA produced from total RNA treated with RNase R previously to reverse transcription. bp: base pairs.

PCR reactions with divergent primers were used in order to validate the back-splicing site of the five circRNAs presenting miRNA binding sites, all with more than two reads in AGO-IP libraries. Only one of the five circRNAs predicted by bioinformatics was amplified by RT-PCR using total RNAs extracted from A. thaliana flowers followed by RNase treatment and divergent primers (Figure 2). The circ_At3g13990 showed the expected electrophoretic band profile of 312 bp (Table 2). PCR negative and positive controls were done using genomic DNA (gDNA) and cDNAs from the parental gene with divergent and convergent primers, respectively. These amplification products were not detected in RNA samples from leaf, silique and steam (data not shown).

**Figure 3:**
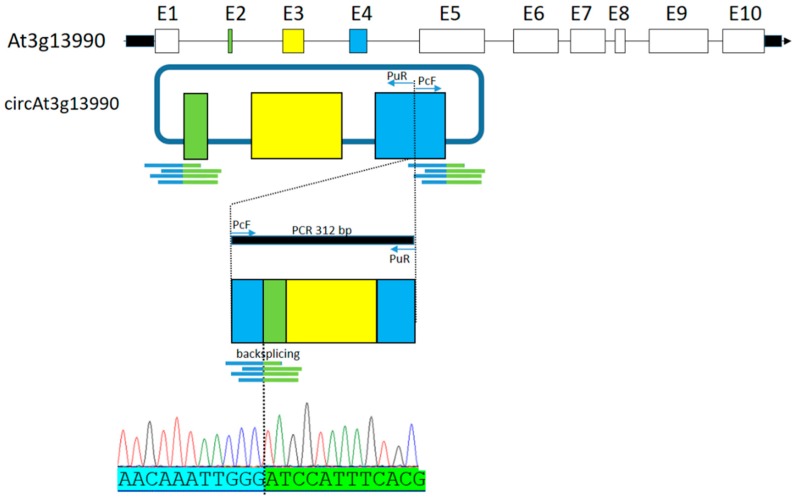
circRNA_At3g13990 back-splicing validation by sequencing. The parental gene structure is represented by exons (boxes), introns (black lines) and 5′ and 3′ untranslated regions (black rectangles). Filled boxes represent exons encompassing the circRNA. Sequencing reactions were performed using PcF and PuR primer. Lines indicated below the colored boxes represent reads matching the back-splicing junction. The nucleotide sequence flanking the back-splicing is represented as an electropherogram. Primer universal Reverse (PuR), Primer circular Forward (PcF) and base pairs (bp).

The total RT-PCR product from circ_At3g1399080 was purified and submitted to Sanger sequencing. The sequence resulted from back-splicing of At3g13990 exon 4 (E4) and exon 2 (E2) was obtained using the Primer circular Forward (PcF) (Figure 3). This result was also corroborated by 34 reads, present in AGO-IP libraries, that overhang with 3 or 4 nucleotides over the back-splicing site.

## 3. Discussion

At present, the role of circular RNA (circRNA) as one of the several classes of competing endogenous RNA (ceRNA) was only demonstrated in animals. They can act as sponges of miRNAs, modulating miRNA action upon target mRNAs. Nevertheless, circRNAs have been identified all across the eukaryotic tree of life [23]. Argonaute (AGO) is an important regulatory protein, with nuclease activity, that is involved in the pathway of RNA-induced silencing. AGO harbors a small RNA in its active site and places it in the correct sequence position in relation to the RNA target in the silencing complex (RISC). The interaction between miRNA-AGO and mRNA target forms a ternary complex and leads to transcripts regulation either by the mRNA cleavage or by translational repression [4]. Another possible molecular component in this ternary complex would be miRNA-AGO and circRNAs. Considering that circRNAs can act as miRNA sponges in mammals [12], publicly available sequencing data from AGO-IP RNAseq libraries were used to screen for circRNAs with this same function in *A. thaliana*. There are studies using the AGO-IP protocol [2,45]. However, because there are no other experiments available with AGO-IP followed by both small RNAseq and RNAseq, we used the data from Carbonell et al. [44], to develop our work.

There are several algorithms available to identify circRNAs. In this work, we used the CirComPara pipeline to detect, quantify and annotate circRNAs from RNA-seq data. This software comprises four different methods for back-splice identification. Each of them has its own features and requirements for the identification. This is the reason why we observed a such variability in the number of identified circRNAs. In order to increase the detection reliability of circRNAs, only those identified by at least 3 methods were selected. These circRNAs present a wide chromosomal distribution, since their loci are on chromosomes 1, 2, 3 and 5 of *A. thaliana*. In addition, their parental genes presented a considerable diversity of functions [46,47]. Using the PlantcircBase, which is a database for plant circular RNAs [48], only circRNA_At1g31810, circRNA_At2g35940, circRNA_At5g16880, circRNA_At3g13990 and circRNA_At5g27720 were previously identified. Besides, they are not conserved between others plant species. Until the present work, none of them had been validated. However, we show the circ_At3g13990 validation, by quantitative PCR and by sequencing. From the 12 circRNAs, only 5 have miRNA binding sites for miRNAs with read counts that are higher in AGO-IP libraries than in the empty vector library.

Interestingly, the majority of enriched miRNAs (6 out 10) have mismatches at the central region of hybridization with their target circRNAs and were predicted as having translation inhibition, which should avoid the degradation of the circRNAs, as observed in mammals’ miRNAs sponges [49]. In this scenario, circRNAs would be able to capture miRNAs for longer periods and increase their sponge activity efficiency, avoiding the negative regulation of miRNAs on their target transcripts. At the same time, the miRNAs: mRNA targets found in AGO-IP libraries were predicted as being inhibited by cleavage (Table 4).

One circRNAs with miRNA binding sites (circ_At3g13990 and circ_At1g12080) was validated by both RT-PCR and sequencing. Except in flower, no amplification products were detected in the other tissues analyzed. This indicates that these circRNAs are tissue specific, a feature also observed in other works [27,50,51]. Besides that, circ_At1g12080 presented more than one amplification product in the electrophoresis analysis. This indicates that different circular isoforms can be produced from a given gene and that their expression can be specific to cell type, tissue, and developmental stage.

The circ_At3g13990 is a perfect case study. It was predicted by 3 methods; the amplified PCR product had the expected size of 312 bp and contains perfect back-splicing site confirmed by Sanger sequencing. RT-PCR performed in RNA samples treated with RNAse R produce the expected 312 bp amplification, with the same intensity as in untreated samples, thereby demonstrating the circular nature of this RNA molecule. The PCR product from the parental cDNA, which originated from RNA samples previously treated with RNase R, revealed a weak amplification. It could indicate that not all RNA was degraded. The circ_At3g13990 contains three predicted miRNA binding sites, but just one of them, miR4239-5p, was significantly more frequently found in AGO-IP libraries. It indicates that this circRNA may be acting as miRNA sponge, blocking the action of miRNA upon its target. Curiously, no mRNA target for miR4239-5p was identified among the reads enriched in the AGO-IP libraries. It suggests that the majority of miR4239-5p molecules are associated to the miRNA:AGO:circRNAs ternary complex. Thus, not allowing the formation of the alternative miRNA:AGO:mRNA complex that would downregulate the target gene expression. It is noteworthy that miR4239-5p has as predicted targets: the small RNA degrading nuclease 3-SDN3 (At5g67240), the UBP1-associated protein 2A (At3g56860) and the gamma tubulin complex protein (At3g43610). All these three gene present a higher expression in flowers and carpels compared to other tissues, according to the BAR eFP Browser from the TAIR database (www.arabidopsis.org).

Our data contributed to the knowledge about the role of circRNAs in plants, since no work until now had demonstrated the existence of a ternary complex formed by AGO:miRNA:circRNA. These findings allow us to propose that plants circRNAs are also able to act as miRNA sponges and modulate the mRNA target regulation by using miRNA.

## 4. Materials and Methods

### 4.1. mRNAseq and Small RNAs Libraries

The RNAseq and AGO-IP small RNAs libraries [44] were downloaded from Gene Expression Omnibus (GEO, accession number GSM989339—GSM989346 and GSM989350—GSM989352) of NCBI. Quality and the presence of adapters in these libraries was visualized using FastQC software (http://www.bioinformatics.babraham.ac.uk/projects/fastqc/). Next, quality trimming and adaptor removal in the small RNAs and RNAseq libraries were carried out using Cutadapt/Sickle (https://github.com/najoshi/sickle) and Trimmomatic [52], respectively.

### 4.2. circRNAs Identification in mRNAseq Libraries from AGO-IP

Clean data from the AGO-IP RNAseq libraries SRR546147, SRR546148, SRR546149 and SRR546150 were used to identify, quantify and annotate potential circRNAs using the CirComPara pipeline [53], which uses five different methods in parallel: FindCirc, TestRealign and CircExplorer2, that works with three different aligners (Segemhel, Star and Tophat). All methods realized back-splice identification in each library with a minimum of 2 reads. *A. thaliana* genome and annotation files obtained from Ensembl Plants (https://plants.ensembl.org/index.html) were used as references. Only circRNAs identified by at least 3 methods were selected for the subsequent analyses.

### 4.3. Analysis of Target mRNAs and miRNAs Counts in AGO-IP and Control Libraries

The psRNATarget tool [54] was used to identify potential miRNAs that could interact with the circRNAs identified in the AGO-IP libraries. All mature miRNAs of *A. thaliana* from miRBase release 21 [55] were used in this analysis. Those miRNAs with an Expectation value (number of mismatches allowed) of 5 or less were selected to subsequent analysis. The Bowtie algorithm [56] was used to align the small RNAs sequences from each library to the miRNA sequences of the selected miRNAs to obtain read count values. The default parameters were used for the alignment and no mismatch was allowed. The miRNAs read counts were normalized according to the size of the libraries. A miRNA was considered to be enriched in the AGO-IP libraries if the normalized read count values of the miRNA were higher in the two AGO-IP libraries compared to the control (input).

The data from the same libraries used for the identification of the circRNAs was used to evaluate if the mRNAs targets of the selected miRNAs were also enriched in the AGO-IP libraries. The putative target mRNAs were selected using the psRNATarget tool. The enriched miRNAs and the transcriptome from *A. thaliana* TAIR version 10 obtained from Phytozome database (https://phytozome.jgi.doe.gov/pz/portal.html) were selected for this analysis using a maximum expectation value of 3.

To obtain the read count value, the reads from each library were mapped against the *A. thaliana* transcriptome using the Bowtie2 algorithm [57] with the default parameters. The DESeq package from the R software [49] was used to identify the target mRNAs significantly more frequent (maximum adjusted *p*-value of 0.05) in the four AGO-IP libraries compared to the input total mRNA controls.

### 4.4. Plant Material and Growth Condition

*A. thaliana* plants of ecotype Columbia were used. After incubation in the dark at 4 °C for 3 days, seeds were cultivated in soil for six weeks, at a temperature of 22 °C and a photoperiod of 16 h of light. Samples of leaves, flowers, axis and siliques were collected and stored at liquid nitrogen for subsequent storage at −80 °C.

### 4.5. RNA Extraction, RNase R Treatment and cDNA Synthesis

The RNA was extracted using the Trizol (Invitrogen) reagent, according to the manufacturer’s instructions. The RNA integrity was performed using 1% agarose gel electrophoresis, where it was visualized under UV light and a digital image generated by the Gel-Doc (Bio-Rad) system. Prior to cDNA synthesis, samples containing 1 g of total RNA were treated with 2 units of RNase R (Lucigen) for 60 min at 37 °C. For cDNA synthesis were used the reverse primer of each analyzed circRNA and the M-MLV Reverse Transcriptase (Promega), according to the manufacturer’s instructions.

### 4.6. Primers Design

Primers were projected using the Primer3 tool [58]. To validate the circRNAs identified by bioinformatic, divergent primers were projected [59]. In order to amplify part of the circRNA we used the primer combination PcF/PuR and to amplify all circRNA sequence we used the primer combination PcF/PuRi. As control, a set of convergent primers were designed for the parental mRNA detection The Reverse universal primer was the same for both circRNA and mRNA detection (Supplementary Table S1).

### 4.7. circRNAs and Parental mRNAs Amplification and Sequencing

The expression of the five circRNAs was evaluated by RT-PCR, using divergent primers. Samples were analyzed in technical triplicates and biological quadruplicates. The Polymerase Chain Reactions (PCR) reactions were realized using the Platinum Taq DNA polymerase (Invitrogen) enzyme. All RT-PCR reactions were performed on the Applied Biosystems Veriti apparatus. PCR conditions were conducted in a volume of 20 μL containing 10 μL of the diluted cDNA (1:100), 0.4 mM dNTPs, 10× Buffer, 3 mM MgCl 2, 0.25 U Platinum Taq DNA polymerase (Invitrogen) and 0.1 μM of each oligonucleotide. PCR conditions were: an initial 2 min step at 95 °C followed by 40 cycles of 10 s denaturing at 95 °C, 15 s annealing at 60 °C and 15 s extension at 72 °C. Confirmation of the fragments was performed by 3% agarose gel electrophoresis. The circRNAs predicted by bioinformatics and confirmed by PCR were purified using the Wizard SV gel PCR clean-up system (Ludwig Biotecnologia) according to the manufacturer’s recommendations. Sanger sequencing reactions were performed with purified PCR products at a final concentration of the reaction of 4.5 pmol/μL, using the PcF, PuR, or PuRi primers (Supplementary Table S1).

## Figures and Tables

**Table 1:** Number of circRNAs identified in AGO-IP libraries by 5 different methods.

Identification Method		CircExplorer2			FindCirc	TestRealign
		Segemehl	Star	Tophat	-	-
CircExplorer2	Segemehl	86	9	12	10	26
	Star	-	15	7	3	3
	Tophat	-	-	23	7	7
FindCirc		-	-	-	1422	198
TestRealign		-	-	-	-	27,812

**Table 2:** Description of 12 putative circRNAs predicted by at least three methods.

Library	Gene_ID	Circ_ID	Parental Gene Function	Origin	Exons	Length (nt) ***	Methods
DDH-IP	At1g02560	circ_At1g02560	Nuclear encoded CLP protease 5	exonic	2	123	5
DDH-IP	At1g12080 **	circ_At1g12080	Vacuolar calcium-binding protein-related	exonic *	1	95	4
DDH-IP	At1g31810	circ_At1g31810	Formin Homology 14	exonic	1	50	3
DDH-IP	At1g52360	circ_At1g52360	Coatomer beta subunit	intronic	1	224	3
DDH-IP	At2g02410	circ_At2g02410	K06962—uncharacterized protein (K06962)	exonic	1	71	4
DDH-IP	At2g35940 **	circ_At2g35940	BEL1-like homeodomain 1	exonic	1	930	4
DDH-IP	At2g42170 **	circ_At2g42170	Actin family protein	exonic	4	1063	5
DDH-IP	At5g16880	circ_At5g16880	Target of Myb protein 1	exonic *	1	49	4
DDH-IP	At5g56950	circ_At5g56950	NAP-1 Nucleosome assembly protein	intronic *	1	118	4
DAH-IP	At3g01800	circ_At3g01800	Ribosome recycling factor	exonic	1	68	4
DAH-IP	At3g13990 **	circ_At3g13990	Kinase-related protein (DUF1296)	exonic	3	349	3
DAH-IP	At5g27720 **	circ_At5g27720	Small nuclear ribonucleoprotein family protein	exonic	4	321	5

* circRNA originated from an imperfect back-splicing; ** circRNAs with miRNA binding site; *** only exons considered.

**Table 3:** Read counts of circRNA and microRNAs that are potentially associated.

circRNA	circRNA Read Counts ***		miRNA	miRNA Read Counts			Inhibiton By
	AGO-IP	Total RNA		AGO1-DDH	AGO1-DAH	Empty Vector	
circ_At1g12080 **	175	13	miR4221-5p *	265	171	7	Cleavage
			miR838-3p *	226	59	41	Translation
-	-	-	miR397a-5p *	1014	520	29	Translation
-	-	-	miR5654-3p	2	0	0	Cleavage
circ_At2g35940	8	0	miR8182-5p *	2	9	0	Translation
-	-	-	miR830-3p *	29	13	0	Cleavage
-	-	-	miR833a-5p *	50	35	17	Translation
-	-	-	miR8174-3p	-	-	-	Cleavage
circ_At2g42170	8	0	miR831-3p *	81	11	3	Translation
-	-	-	miR838-3p *	226	59	41	Cleavage
-	-	-	miR4239-5p *	17	7	0	Translation
circ_At3g13990 **	17	0	miR5637-5p	-	-	-	Cleavage
-	-	-	miR780.2-3p	-	-	-	Cleavage
circ_At5g27720	17	0	miR838-3p *	226	59	41	Cleavage

* miRNA considered enriched in AGO-IP libraries; ** circRNAs validated; *** Read count normalized by the library size and with difference between assembled AGO-IPs (AGO-DAH/DDH) and Input libraries (*p* < 0.05); Expectation value ≤5.

**Table 4:** mRNAs targeted by miRNAs with circRNAs and enriched in AgoIP libraries.

Target_Access	miRNA	Expectation	Inhibition By	Lenght	Target Counts *		Function
					AgoIP	Input	
At2g38080.1	miR397a-5p	1	Cleavage	2021	58	21	Laccase/Diphenol oxidase
At5g60020.1		1	Cleavage	2049	33	18	Laccase 17
At3g06040.1		3	Cleavage	864	29	14	Ribosomal protein L12
At3g06470.1		3	Cleavage	1092	75	4	GNS1/SUR4 membrane protein
At3g54170.1	miR4221-5p	2.5	Cleavage	1262	22	10	FKBP12 interacting protein 37
At4g13070.1		2.5	Cleavage	1775	8	2	RNA-binding CRS1
At5g60040.1		2.5	Cleavage	4582	62	22	Nuclear RNA polymerase C1
At1g13350.1		3	Cleavage	2454	142	24	Protein kinase
At1g77660.1		3	Cleavage	1765	22	12	H3K4-specific methyltransferase
At2g33240.1		3	Cleavage	5313	36	12	Myosin XI D
At3g02170.1		3	Cleavage	3300	319	155	Longifolia2
At4g14510.1		3	Cleavage	2940	57	22	CRM family member 3B
At1g31650.1		3	Translation	2255	164	28	RHO guanyl- exchange factor 14
At2g38610.1		3	Translation	1452	56	26	RNA-binding KH protein
At2g35160.1	miR8182-5p	3	Cleavage	2798	20	9	SU(VAR)3-9 homolog 5
At4g22580.1		3	Cleavage	1628	39	10	Exostosin family protein
At1g23400.1		3	Cleavage	1822	81	24	RNA-binding CRS1
At1g49880.1	miR831-3p	2.5	Translation	803	50	2	FAD-linked sulfhydryl oxidase
At3g46060.1		3	Translation	1132	75	41	RAS-related protein RABE1C
At2g36890.1	miR833a-5p	2.5	Cleavage	971	6	1	Myb-like DNA-binding domain
At3g12380.1	miR838-3p	2.5	Cleavage	2323	33	16	Actin-related protein 5
At1g21740.1		3	Cleavage	2862	63	22	Protein of unknown function
At1g64180.1		3	Cleavage	2072	13	3	Intracellular transport protein
At1g70470.1		3	Cleavage	765	17	4	No annotated domains
At4g01080.1		3	Cleavage	1583	98	33	Trichome-birefringence like 26
At5g09460.1		3	Cleavage	2546	124	41	Transcription Factor SAC51
At5g09461.1		3	Cleavage	2546	124	41	Conserved peptide upstream ORF
At5g20110.1		3	Cleavage	778	28	2	Dynein light chain type 1
At5g46030.1		2	Translation	732	26	12	Elongation factor Elf1 like
At2g44430.1		2.5	Translation	2196	98	19	DNA-binding protein
At5g22640.1		2.5	Translation	2814	247	115	MORN repeat-containing protein
At5g40340.1		2.5	Translation	3096	624	75	Tudor/PWWP/MBT protein
At5g56210.1		2.5	Translation	2004	22	5	WPP domain interacting protein 2
At5g62390.1		2.5	Translation	1859	349	152	BCL-2-associated athanogene 7
At5g17910.1		3	Translation	4532	178	77	No annotated domains
At5g41960.1		3	Translation	874	9	4	No annotated domains
At5g57790.1		3	Translation	1407	29	12	No annotated domains

* Read count normalized by the library size and with difference between assembled AGO-IPs (AGO-DAH/DDH) and Input libraries (*p* < 0.05).

## Supplementary Materials

The following are available online at https://www.mdpi.com/2223-7747/8/9/302/s1, Table S1: Primers used for PCR validation; Table S2: Description of total targets of miRNAs with sites in circRNAs; Table S3: Location of circRNAs identified in at least three methods.
