# Supplementary material for: Circular and Micro RNAs from Arabidopsis thaliana Flowers Are Simultaneously Isolated from AGO-IP Libraries
Source: Plants (Basel). 2019 Aug 26;8(9):302. doi: 10.3390/plants8090302 (PMC6784238; doi:10.3390/plants8090302)
Supplement: Supplementary file 1 [file plants-08-00302-s001.pdf]

Supplementary Table 1. Primers used for PCR validation

Primer name	Sequence (5' → 3')
PCF_At2g42170	GCAGCCGCTAATATGAGG
PUR_At2g42170	GTTTTCGCCCTCTACTTCC
PPF_At2g42170	GAGCTGTCAGCAGCATTC
PCFi_At2g42170	TGGAGAAGATGCACCAAGTG
PCRi_At2g42170	CTCTTCAATCCCTCGGAACA
PCF_At1g12080	AATTGTAGAAACAAACGAAGAAGT
PUR_At1g12080	GGTGCTTCGGATTTTTGTTC
PPF_At1g12080	GAAATGGCCACCGTTGAG
PCFi_At1g12080	CCGAGAAGAAAACAGAGGAGAA
PCRi_At1g12080	CTGCCTCTTCCACAACCACT
PCF_At2g35940	GGCACGGCAGAGAGAC
PUR_At2g35940	CGACGTCGTTTTCTTTGATTC
PPF_At2g35940	GCAATCACACACATCTCCAC
PCFi_At3g25940	GCCGACAACATCTCCGTACT
PCRi_At2g35940	CGATGATTCTCCGACAGGTT
PCF_At5g27720	AAGAGGACGTGGTGTGG
PUR_At5g27720	TTCACCAAATGCCCATTGTA
PPF_At5g27720	GTTCACCTTCTTGCCGATC
PCFi_At5g27720	ATGAACATCCATCTGCGTGA
PCRi_At5g27720	CCAACACCTGGTGGTTTTCT
PCF_At3g13990	ATTCTTCTGCCCAGTCCTC
PUR_At3g13990	TCTTTATGTTGGCAGGCTC
PPF_At3g13990	GCTAACGAGGCTGTGGA
PCFi_At3g13990	GGAATTTCTTCCCCAGGAAC
PCRi_At3g13990	TCCCTAACAACGCGAAACTC

PCF: Primer Circular Foward

PUR: Primer Univeral Reverse

PPF: Primer parental Foward

PCFi: Primer Circular Intern

PCRi: Primer Circular Reverse Intern

Supplementary Table 2. Description of total targets of miRNAs with sites in circRNAs

	target_Access	miRNA	Expectation	Inhibition by	Lenght	Target counts										Target counts					
						DDH_WT1	DDH_WT2	DAH_mut1	DAH_mut2	DDH_input_WT1	DDH_input_WT2	DAH_input_mut1	DAH_input_mut2	Ago-IP	Input	AgoIP (norm)				Input (n	
						13,85	4,63	9,05	10,64	3,54	1,00	2,88	1,99								
	At2g29130.1		0,5	Cleavage	2070	73	33	22	10	12	0	3	1	35	4	5	7	2	1	3	0
*	At2g38080.1		1	Cleavage	2021	1117	260	417	507	79	12	73	50	575	54	81	56	46	48	22	12
**	At5g60020.1		1	Cleavage	2049	523	169	288	285	78	11	49	41	316	45	38	37	32	27	22	11
	At1g66670.1		2,5	Cleavage	1196	443	189	370	454	143	36	134	67	364	95	32	41	41	43	40	36
	At3g60250.1		2,5	Cleavage	1359	252	94	154	148	90	28	89	41	162	62	18	20	17	14	25	28
	At3g60250.2		2,5	Cleavage	1356	244	91	161	147	91	30	87	41	161	62	18	20	18	14	26	30
*	At3g06040.1		3	Cleavage	864	406	174	217	288	47	16	46	24	271	33	29	38	24	27	13	16
*	At3g06040.2	miR397a	3	Cleavage	1253	406	174	217	288	47	18	49	25	271	35	29	38	24	27	13	18
*	At3g06040.3		3	Cleavage	657	395	162	202	270	41	15	45	23	257	31	29	35	22	25	12	15
*	At3g06470.1		3	Cleavage	1092	645	229	979	1038	15	2	23	7	723	12	47	50	108	98	4	2
	At4g33230.1		3	Cleavage	1830	86	11	124	83	13	2	18	11	76	11	6	2	14	8	4	2
	At5g11210.1		3	Cleavage	2490	0	0	3	0	2	0	0	0	1	1	0	0	0	0	1	0
	At2g05170.1		3	Translation	3334	688	217	398	561	212	46	135	113	466	127	50	47	44	53	60	46
	At2g28940.1		3	Translation	1591	72	30	36	56	21	11	12	10	49	14	5	6	4	5	6	11
	At2g28940.2		3	Translation	1539	72	30	36	56	21	11	12	10	49	14	5	6	4	5	6	11
	At5g64020.1		3	Translation	1683	173	59	115	124	39	12	38	26	118	29	12	13	13	12	11	12
	At1g69740.1		2,5	Cleavage	1680	1097	320	645	779	492	140	320	277	710	307	79	69	71	73	139	140
	At1g69740.2		2,5	Cleavage	1625	1015	298	579	645	490	139	321	277	634	307	73	64	64	61	138	139
	At1g76160.1		2,5	Cleavage	2008	912	331	374	575	326	67	521	208	548	281	66	72	41	54	92	67
	At2g43980.1	miR4221	2,5	Cleavage	1691	94	21	54	82	45	9	57	27	63	35	7	5	6	8	13	9
*	At3g54170.1		2,5	Cleavage	1262	387	131	152	172	41	5	27	26	211	25	28	28	17	16	12	5
*	At4g13070.1		2,5	Cleavage	1775	173	25	50	92	10	1	8	3	85	6	12	5	6	9	3	1
	At4g15660.1		2,5	Cleavage	505	0	0	14	10	2	0	3	0	6	1	0	0	2	1	1	0

	At4g15670.1	2,5	Cleavage	587	0	0	0	11	1	0	2	0	3	1	0	0	0	1	0	0
*	At5g60040.1	2,5	Cleavage	4582	1060	279	512	582	78	28	39	45	608	48	77	60	57	55	22	28
*	At5g60040.2	2,5	Cleavage	4428	792	180	308	285	71	25	33	43	391	43	57	39	34	27	20	25
	At5g60310.1	2,5	Cleavage	1851	5	2	0	0	3	0	2	2	2	2	0	0	0	0	1	0
	At5g64710.1	2,5	Cleavage	2771	142	36	51	60	22	11	11	17	72	15	10	8	6	6	6	11
	At5g64710.2	2,5	Cleavage	2294	54	27	28	59	20	8	10	15	42	13	4	6	3	6	6	8
*	At1g13350.1	3	Cleavage	2454	3047	817	812	858	100	17	75	52	1384	61	220	177	90	81	28	17
*	At1g13350.2	3	Cleavage	2535	3062	817	814	874	101	17	77	51	1392	62	221	177	90	82	28	17
	At1g20500.1	3	Cleavage	1721	16	1	0	0	1	1	0	0	4	1	1	0	0	0	0	1
	At1g34150.1	3	Cleavage	1735	87	30	78	86	27	11	21	19	70	20	6	6	9	8	8	11
	At1g64000.1	3	Cleavage	888	3	0	0	6	0	0	0	0	2	0	0	0	0	1	0	0
	At1g69930.1	3	Cleavage	1048	1	0	4	0	3	0	2	1	1	2	0	0	0	0	1	0
	At1g77350.1	3	Cleavage	813	197	75	80	80	32	4	29	21	108	22	14	16	9	8	9	4
	At1g77350.2	3	Cleavage	838	175	64	80	61	32	4	26	19	95	20	13	14	9	6	9	4
**	At1g77660.1	3	Cleavage	1765	336	82	208	242	32	10	50	25	217	29	24	18	23	23	9	10
	At2g23630.1	3	Cleavage	1647	0	0	0	0	1	0	0	2	0	1	0	0	0	0	0	0
*	At2g33240.1	3	Cleavage	5313	803	210	250	149	42	6	32	34	353	29	58	45	28	14	12	6
*	At3g02170.1	3	Cleavage	3300	5636	1840	2274	2338	569	93	588	325	3022	394	407	398	251	220	161	93
	At3g05310.1	3	Cleavage	1947	6	0	6	0	1	0	0	1	3	1	0	0	1	0	0	0
*	At4g14510.1	3	Cleavage	2940	1136	317	304	474	89	22	51	47	558	52	82	69	34	45	25	22
	At4g19850.2	3	Cleavage	663	10	7	1	3	1	0	1	0	5	1	1	2	0	0	0	0
	At5g20320.1	3	Cleavage	5660	641	225	321	419	234	45	165	97	402	135	46	49	35	39	66	45
	At5g20320.2	3	Cleavage	5648	632	222	321	410	229	45	162	95	396	133	46	48	35	39	65	45
	At5g48655.1	3	Cleavage	1132	119	37	70	62	73	10	57	34	72	44	9	8	8	6	21	10
	At5g48655.2	3	Cleavage	1105	128	42	102	68	73	10	59	34	85	44	9	9	11	6	21	10
	At5g48655.3	3	Cleavage	1448	128	41	102	68	73	10	59	34	85	44	9	9	11	6	21	10
	At5g66710.1	3	Cleavage	1448	7	5	0	5	4	0	3	1	4	2	1	1	0	0	1	0
	At2g46770.1	2	Translation	1382	150	62	22	22	6	0	14	5	64	6	11	13	2	2	2	0
	At5g54410.1	2,5	Translation	660	0	0	0	0	1	0	0	0	0	0	0	0	0	0	0	0
**	At1g22230.1	3	Translation	1262	34	15	14	4	0	0	2	0	17	1	2	3	2	0	0	0
*	At1g31650.1	3	Translation	2255	1775	661	1602	2222	128	19	59	72	1565	70	128	143	177	209	36	19
	At1g55930.1	3	Translation	2239	106	61	33	106	35	9	17	20	77	20	8	13	4	10	10	9

	At1g58150.1		3	Translation	276	29	11	4	22	3	1	0	5	17	2	2	2	0	2	1	1
*	At2g38610.1		3	Translation	1452	791	266	573	496	76	21	89	60	532	62	57	58	63	47	21	21
*	At2g38610.2		3	Translation	1450	801	273	583	500	76	21	90	60	539	62	58	59	64	47	21	21
	At2g40760.1		3	Translation	1526	210	69	91	204	39	12	23	24	144	25	15	15	10	19	11	12
	At2g45060.1		3	Translation	1229	175	39	122	105	41	7	47	35	110	33	13	8	13	10	12	7
	At3g14200.1		3	Translation	1292	201	71	52	106	86	19	92	43	108	60	15	15	6	10	24	19
	At5g65020.1		3	Translation	1226	328	104	185	224	164	34	152	103	210	113	24	22	20	21	46	34
	At5g65020.2		3	Translation	1084	234	83	161	144	160	31	149	100	156	110	17	18	18	14	45	31
	At5g14860.1		2	Cleavage	1479	10	5	0	20	2	0	0	2	9	1	1	1	0	2	1	0
	At3g43610.1		2,5	Cleavage	3876	792	247	362	450	143	35	108	90	463	94	57	53	40	42	40	35
	At2g28250.1		3	Cleavage	2066	173	35	65	127	23	10	23	20	100	19	12	8	7	12	6	10
	At2g28250.2	miR4239	3	Cleavage	2007	181	35	65	127	23	10	22	20	102	19	13	8	7	12	6	10
	At4g25170.1		3	Cleavage	1391	257	86	196	181	110	19	76	49	180	64	19	19	22	17	31	19
	At4g25170.2		3	Cleavage	1436	248	86	196	181	110	19	74	49	178	63	18	19	22	17	31	19
	At5g67240.1		3	Cleavage	2639	707	244	381	446	95	37	77	78	445	72	51	53	42	42	27	37
	At5g67240.2		3	Cleavage	2391	631	232	349	409	84	33	74	72	405	66	46	50	39	38	24	33
*	At2g35160.1		3	Cleavage	2798	354	108	163	129	48	6	23	17	189	24	26	23	18	12	14	6
	At3g19720.1		3	Cleavage	2678	843	217	520	658	179	42	146	115	560	121	61	47	57	62	51	42
	At3g19720.2		3	Cleavage	2570	774	205	503	635	174	40	146	112	529	118	56	44	56	60	49	40
	At3g19720.3		3	Cleavage	2678	767	202	484	617	160	36	144	106	518	112	55	44	53	58	45	36
	At3g61028.1		3	Cleavage	977	0	1	0	0	2	0	1	0	0	1	0	0	0	0	1	0
	At3g61028.2		3	Cleavage	980	1	3	0	9	2	0	1	0	3	1	0	1	0	1	1	0
*	At4g22580.1		3	Cleavage	1628	644	231	326	264	33	3	41	27	366	26	47	50	36	25	9	3
	At4g39690.1	miRNA8182	3	Cleavage	2390	511	161	273	330	110	23	74	57	319	66	37	35	30	31	31	23
	At5g27030.1		3	Cleavage	3655	900	364	522	868	232	60	170	120	664	146	65	79	58	82	65	60
	At5g27030.2		3	Cleavage	3698	887	364	517	837	232	60	171	119	651	146	64	79	57	79	65	60
	At5g27630.1		3	Cleavage	2247	156	61	78	239	41	3	21	28	134	23	11	13	9	22	12	3
	At3g61280.1		2	Translation	1976	7	0	0	0	0	0	0	0	2	0	1	0	0	0	0	0
	At3g61280.2		2	Translation	2079	7	0	0	0	0	0	0	0	2	0	1	0	0	0	0	0
	At2g35580.1		3	Translation	1125	0	0	0	0	0	0	0	0	0	0	0	0	0	0	0	0
	At4g15740.1		3	Translation	1574	0	0	0	0	0	0	0	0	0	0	0	0	0	0	0	0
	At2g28150.1	miR830	3	Translation	2074	224	78	103	169	60	28	34	43	144	41	16	17	11	16	17	28

	At5g49130.1		2,5	Cleavage	1541	472	146	288	329	81	30	53	35	309	50	34	32	32	31	23	30
*	At1g23400.1		3	Cleavage	1822	940	351	857	926	83	22	54	64	769	56	68	76	95	87	23	22
*	At1g49880.1		2,5	Translation	803	588	170	550	622	4	0	4	8	483	4	42	37	61	58	1	0
	At1g69980.1		2,5	Translation	841	122	44	87	136	21	2	29	10	97	16	9	10	10	13	6	2
	At5g08560.1		2,5	Translation	2299	863	255	486	493	170	40	110	77	524	99	62	55	54	46	48	40
	At5g08560.2	miR831	2,5	Translation	2226	865	251	476	483	168	40	110	77	519	99	62	54	53	45	47	40
	At1g23360.2		3	Translation	1303	86	26	31	61	20	8	27	19	51	19	6	6	3	6	6	8
	At3g14190.1		3	Translation	903	123	22	78	95	55	19	24	22	80	30	9	5	9	9	16	19
*	At3g46060.1		3	Translation	1132	1051	331	689	824	129	31	156	86	724	101	76	72	76	77	36	31
*	At3g46060.2		3	Translation	1142	937	292	648	776	123	29	147	82	663	95	68	63	72	73	35	29
*	At3g46060.3		3	Translation	1061	938	289	621	760	124	29	147	82	652	96	68	62	69	71	35	29
	At5g55280.1		3	Translation	1512	398	121	330	401	112	36	108	52	313	77	29	26	36	38	32	36
	At1g09730.1		2,5	Cleavage	3386	553	225	400	312	144	35	135	121	373	109	40	49	44	29	41	35
	At1g09730.2	miR833a	2,5	Cleavage	3287	540	225	400	312	147	35	137	121	369	110	39	49	44	29	41	35
**	At2g36890.1		2,5	Cleavage	971	101	28	26	62	6	0	7	2	54	4	7	6	3	6	2	0
	At4g34170.1		3	Cleavage	882	8	0	0	0	0	0	0	0	2	0	1	0	0	0	0	0
	At1g23020.1		2	Cleavage	2252	126	62	224	231	36	6	30	18	161	23	9	13	25	22	10	6
	At1g23020.2		2	Cleavage	2271	126	62	226	231	36	6	30	18	161	23	9	13	25	22	10	6
*	At1g67230.1		2,5	Cleavage	3981	6754	2694	1912	2013	467	113	413	291	3343	321	488	582	211	189	132	113
**	At4g15410.1		2,5	Cleavage	1542	724	254	385	405	77	24	72	73	442	62	52	55	43	38	22	24
	At5g18590.1		2,5	Cleavage	2719	429	190	274	323	152	33	139	99	304	106	31	41	30	30	43	33
	At5g18590.2		2,5	Cleavage	2701	429	190	274	323	152	33	139	99	304	106	31	41	30	30	43	33
	At1g01160.1		3	Cleavage	1045	900	277	611	656	76	19	81	44	611	55	65	60	68	62	21	19
	At1g01160.2	miR837	3	Cleavage	1129	878	266	587	634	71	20	79	43	591	53	63	58	65	60	20	20
*	At1g01590.1		3	Cleavage	2115	277	86	117	178	21	3	25	16	165	16	20	19	13	17	6	3
	At1g13330.1		3	Cleavage	1020	20	4	34	8	8	1	1	0	17	3	1	1	4	1	2	1
	At1g16800.1		3	Cleavage	6384	727	195	300	317	94	24	69	67	385	64	53	42	33	30	27	24
*	At1g52500.1		3	Cleavage	1721	16	1	0	0	1	1	0	0	4	1	1	0	0	0	0	1
*	At1g52500.2		3	Cleavage	1491	557	138	325	230	36	11	31	30	313	27	40	30	36	22	10	11
	At1g52590.1		3	Cleavage	776	225	56	37	50	21	6	22	8	92	14	16	12	4	5	6	6
	At2g04842.1		3	Cleavage	1953	657	222	356	233	136	45	105	79	367	91	47	48	39	22	38	45
	At2g17620.1		3	Cleavage	1612	64	22	16	37	16	3	9	3	35	8	5	5	2	3	5	3

	At2g32630.1	3	Cleavage	1875	202	57	184	159	5	2	10	5	151	6	15	12	20	15	1	2
	At2g37760.1	3	Cleavage	1247	183	71	60	78	72	27	94	46	98	60	13	15	7	7	20	27
	At2g37760.2	3	Cleavage	1179	183	71	60	78	72	27	94	46	98	60	13	15	7	7	20	27
	At2g37760.3	3	Cleavage	1126	181	68	53	78	69	27	88	43	95	57	13	15	6	7	19	27
	At2g37760.4	3	Cleavage	1255	181	70	53	78	69	27	88	43	96	57	13	15	6	7	19	27
	At2g37760.5	3	Cleavage	1251	181	70	53	78	69	27	88	43	96	57	13	15	6	7	19	27
	At2g47750.1	3	Cleavage	2155	136	40	58	127	54	15	23	34	90	32	10	9	6	12	15	15
*	At3g02950.1	3	Cleavage	1135	212	87	228	184	36	8	25	17	178	22	15	19	25	17	10	8
	At3g07740.1	3	Cleavage	1911	266	63	173	91	36	7	30	19	148	23	19	14	19	9	10	7
	At3g07740.2	3	Cleavage	1997	259	63	173	91	36	7	30	19	147	23	19	14	19	9	10	7
	At3g07740.3	3	Cleavage	1816	246	56	152	91	32	5	28	15	136	20	18	12	17	9	9	5
	At3g07740.4	3	Cleavage	1884	255	60	156	91	35	7	30	19	141	23	18	13	17	9	10	7
*	At3g55860.1	3	Cleavage	1366	2523	784	3148	3402	81	5	81	32	2464	50	182	169	348	320	23	5
	At3g62260.1	3	Cleavage	1571	114	21	40	70	148	8	40	55	61	63	8	5	4	7	42	8
	At3g62260.2	3	Cleavage	1560	97	18	35	70	138	6	37	55	55	59	7	4	4	7	39	6
*	At4g00850.1	3	Cleavage	1062	328	92	204	299	14	2	2	11	231	7	24	20	23	28	4	2
	At4g31150.1	3	Cleavage	1282	123	43	70	65	17	6	5	8	75	9	9	9	8	6	5	6
	At4g31150.2	3	Cleavage	1287	119	47	55	60	16	6	5	7	70	9	9	10	6	6	5	6
	At4g33620.1	3	Cleavage	2352	108	44	87	113	15	2	11	15	88	11	8	10	10	11	4	2
	At5g25410.1	3	Cleavage	1204	1	0	2	0	1	0	0	0	1	0	0	0	0	0	0	0
	At5g42770.1	3	Cleavage	892	42	19	28	23	24	7	19	15	28	16	3	4	3	2	7	7
	At5g42770.2	3	Cleavage	1028	42	19	28	23	25	8	19	15	28	17	3	4	3	2	7	8
	At5g46470.1	3	Cleavage	4849	992	341	469	410	338	32	138	133	553	160	72	74	52	39	95	32
	At5g60370.1	3	Cleavage	1535	178	33	112	52	9	6	16	14	94	11	13	7	12	5	3	6
	At5g61040.1	3	Cleavage	2435	93	34	69	108	37	12	14	11	76	19	7	7	8	10	10	12
**	At5g48600.1	2	Translation	3796	853	309	378	328	103	21	78	66	467	67	62	67	42	31	29	21
*	At5g48600.2	2	Translation	3965	940	321	411	376	109	22	79	68	512	70	68	69	45	35	31	22
	At3g13910.1	2,5	Translation	565	53	13	15	28	10	1	4	1	27	4	4	3	2	3	3	1
*	At1g12430.1	3	Translation	3216	996	486	750	741	159	39	144	83	743	106	72	105	83	70	45	39
*	At1g12430.2	3	Translation	3224	988	476	736	732	157	39	143	83	733	106	71	103	81	69	44	39
	At2g41690.1	3	Translation	735	0	0	0	0	1	0	0	3	0	1	0	0	0	0	0	0
	At4g37820.1	3	Translation	2086	2674	760	1104	1303	89	26	65	45	1460	56	193	164	122	123	25	26

	At5g13500.1		3	Translation	1583	231	63	117	162	33	20	37	30	143	30	17	14	13	15	9	20
	At5g13500.2		3	Translation	1497	193	53	102	151	32	20	34	29	125	29	14	11	11	14	9	20
	At5g13500.3		3	Translation	1453	193	53	102	151	32	20	34	29	125	29	14	11	11	14	9	20
	At5g58610.1		3	Translation	3198	17	9	2	62	9	4	6	3	23	6	1	2	0	6	3	4
	At1g62480.1		2	Cleavage	764	3133	1061	2355	2971	1679	305	677	669	2380	833	226	229	260	279	474	305
	At1g09730.2		2,5	Cleavage	3287	540	225	400	312	147	35	137	121	369	110	39	49	44	29	41	35
	At1g10740.1		2,5	Cleavage	1890	799	222	529	398	127	24	114	60	487	81	58	48	58	37	36	24
	At1g10740.2		2,5	Cleavage	1944	796	213	524	405	124	22	113	60	485	80	57	46	58	38	35	22
	At1g10740.3		2,5	Cleavage	1882	766	210	510	366	127	24	113	60	463	81	55	45	56	34	36	24
	At1g10740.4		2,5	Cleavage	1874	766	210	510	361	126	24	113	60	462	81	55	45	56	34	36	24
	At1g36925.1		2,5	Cleavage	525	0	0	0	0	0	0	0	0	0	0	0	0	0	0	0	0
	At1g72360.1		2,5	Cleavage	1016	0	6	8	2	10	2	7	1	4	5	0	1	1	0	3	2
	At1g72360.2		2,5	Cleavage	931	0	6	10	4	10	3	7	1	5	5	0	1	1	0	3	3
	At1g72360.3		2,5	Cleavage	930	0	6	8	2	10	2	7	1	4	5	0	1	1	0	3	2
	At2g35920.1		2,5	Cleavage	3371	568	175	268	316	116	28	96	58	332	75	41	38	30	30	33	28
	At3g07400.1		2,5	Cleavage	3373	396	153	328	198	88	23	84	52	269	62	29	33	36	19	25	23
*	At3g12380.1		2,5	Cleavage	2323	496	188	291	241	65	14	34	43	304	39	36	41	32	23	18	14
*	At3g12380.2	miR838	2,5	Cleavage	2313	496	185	291	241	65	14	34	43	303	39	36	40	32	23	18	14
	At4g22745.1		2,5	Cleavage	844	101	36	82	37	35	19	36	30	64	30	7	8	9	3	10	19
	At4g30720.1		2,5	Cleavage	2488	477	169	329	549	115	52	84	69	381	80	34	37	36	52	32	52
	At5g07400.1		2,5	Cleavage	3564	86	13	75	98	16	3	10	5	68	9	6	3	8	9	5	3
*	At1g21740.1		3	Cleavage	2862	995	449	395	406	57	32	51	48	561	47	72	97	44	38	16	32
*	At1g64180.1		3	Cleavage	2072	209	59	115	127	14	0	12	9	128	9	15	13	13	12	4	0
	At1g69870.1		3	Cleavage	2167	2017	575	1165	1605	553	124	718	305	1341	425	146	124	129	151	156	124
*	At1g70470.1		3	Cleavage	765	237	82	194	143	16	3	12	8	164	10	17	18	21	13	5	3
	At1g75430.1		3	Cleavage	966	0	0	21	13	3	0	3	0	9	2	0	0	2	1	1	0
	At2g19410.1		3	Cleavage	2406	0	0	0	0	0	0	0	0	0	0	0	0	0	0	0	0
	At2g45720.1		3	Cleavage	2039	96	42	50	99	40	5	26	23	72	24	7	9	6	9	11	5
	At2g45720.2		3	Cleavage	2226	96	45	50	100	41	5	26	22	73	24	7	10	6	9	12	5
	At3g26850.1		3	Cleavage	1461	146	64	85	132	25	1	26	18	107	18	11	14	9	12	7	1
	At3g26850.2		3	Cleavage	1257	138	62	77	115	25	1	23	16	98	16	10	13	9	11	7	1
	At3g55140.1		3	Cleavage	1247	216	53	96	102	28	16	48	28	117	30	16	11	11	10	8	16

	At3g55140.2	3	Cleavage	1403	214	46	90	78	26	16	44	26	107	28	15	10	10	7	7	16
*	At4g01080.1	3	Cleavage	1583	1899	587	636	597	125	20	111	80	930	84	137	127	70	56	35	20
	At4g33620.1	3	Cleavage	2352	108	44	87	113	15	2	11	15	88	11	8	10	10	11	4	2
	At4g35440.1	3	Cleavage	2561	381	120	214	277	131	33	42	60	248	67	28	26	24	26	37	33
	At4g35440.2	3	Cleavage	2661	351	111	214	271	128	33	39	57	237	64	25	24	24	25	36	33
	At5g02010.1	3	Cleavage	2133	157	40	106	162	34	6	22	11	116	18	11	9	12	15	10	6
*	At5g09460.1	3	Cleavage	2546	1636	707	1026	1179	198	26	99	93	1137	104	118	153	113	111	56	26
*	At5g09461.1	3	Cleavage	2546	1636	707	1026	1179	198	26	99	93	1137	104	118	153	113	111	56	26
*	At5g09462.1	3	Cleavage	2546	1636	707	1026	1179	198	26	99	93	1137	104	118	153	113	111	56	26
*	At5g09463.1	3	Cleavage	2546	1636	707	1026	1179	198	26	99	93	1137	104	118	153	113	111	56	26
*	At5g20110.1	3	Cleavage	778	440	130	308	209	10	1	8	4	272	6	32	28	34	20	3	1
	At1g51630.1	2	Translation	1652	277	66	137	210	98	31	106	78	173	78	20	14	15	20	28	31
	At2g18490.1	2	Translation	756	0	0	0	0	0	0	0	0	0	0	0	0	0	0	0	0
*	At5g46030.1	2	Translation	732	362	83	220	370	48	16	28	16	259	27	26	18	24	35	14	16
	At1g43170.3	2,5	Translation	1447	5066	1734	3401	4181	2283	847	1890	1456	3596	1619	366	375	376	393	644	847
	At1g67040.1	2,5	Translation	3101	309	91	202	196	44	14	39	28	200	31	22	20	22	18	12	14
	At1g67230.1	2,5	Translation	3981	6754	2694	1912	2013	467	113	413	291	3343	321	488	582	211	189	132	113
	At1g68670.1	2,5	Translation	1583	271	127	103	151	107	16	80	61	163	66	20	27	11	14	30	16
	At2g04840.1	2,5	Translation	1170	13	2	0	0	0	0	1	0	4	0	1	0	0	0	0	0
	At2g33360.1	2,5	Translation	2571	310	121	273	350	98	28	72	42	264	60	22	26	30	33	28	28
	At2g33360.2	2,5	Translation	1922	245	95	214	292	79	22	52	30	212	46	18	21	24	27	22	22
*	At2g44430.1	2,5	Translation	2196	1858	645	595	549	57	20	55	41	912	43	134	139	66	52	16	20
	At3g09490.1	2,5	Translation	1115	8	1	23	0	2	0	0	2	8	1	1	0	3	0	1	0
	At4g10770.1	2,5	Translation	2625	488	137	283	262	66	12	80	33	293	48	35	30	31	25	19	12
	At4g18780.1	2,5	Translation	3251	243	81	193	178	85	24	64	55	174	57	18	18	21	17	24	24
	At4g22200.1	2,5	Translation	3169	887	220	302	308	201	38	120	128	429	122	64	48	33	29	57	38
	At4g37850.1	2,5	Translation	1315	0	0	0	0	0	0	0	0	0	0	0	0	0	0	0	0
	At4g39060.1	2,5	Translation	1107	0	0	0	0	0	0	0	0	0	0	0	0	0	0	0	0
	At4g40020.1	2,5	Translation	1848	148	31	39	13	6	3	6	5	58	5	11	7	4	1	2	3
	At5g20030.1	2,5	Translation	1368	32	13	10	57	6	0	4	6	28	4	2	3	1	5	2	0
*	At5g22640.1	2,5	Translation	2814	3711	1118	2104	2595	447	102	299	258	2382	277	268	242	233	244	126	102
*	At5g22640.2	2,5	Translation	2457	2761	836	1493	1883	375	83	247	213	1743	230	199	181	165	177	106	83

	At5g28350.1	2,5	Translation	3711	982	309	582	723	275	65	203	202	649	186	71	67	64	68	78	65	
*	At5g40340.1	2,5	Translation	3096	12393	3821	4055	3483	261	49	272	166	5938	187	895	826	448	327	74	49	
	At5g41310.1	2,5	Translation	2886	20	6	0	23	10	0	2	1	12	3	1	1	0	2	3	0	
	At5g43130.1	2,5	Translation	2650	1827	689	852	998	139	39	133	98	1092	102	132	149	94	94	39	39	
	At5g43130.2	2,5	Translation	2747	1825	684	844	995	140	37	130	97	1087	101	132	148	93	94	40	37	
*	At5g56210.1	2,5	Translation	2004	299	98	229	203	20	6	7	10	207	11	22	21	25	19	6	6	
	At5g62290.1	2,5	Translation	930	41	37	51	59	55	16	41	35	47	37	3	8	6	6	16	16	
	At5g62290.2	2,5	Translation	927	37	35	46	59	52	16	36	31	44	34	3	8	5	6	15	16	
*	At5g62390.1	2,5	Translation	1859	5667	2041	2685	2662	507	142	423	349	3264	355	409	441	297	250	143	142	
	At5g65300.1	2,5	Translation	625	32	17	10	14	74	4	36	28	18	36	2	4	1	1	21	4	
	At2g01720.1	3	Translation	1719	390	106	496	356	230	54	179	116	337	145	28	23	55	33	65	54	
	At2g36790.1	3	Translation	1677	211	93	237	31	62	11	50	42	143	41	15	20	26	3	17	11	
	At3g12860.1	3	Translation	1608	184	69	126	75	19	6	21	21	114	17	13	15	14	7	5	6	
	At3g16560.1	3	Translation	2166	180	64	112	116	85	6	52	27	118	43	13	14	12	11	24	6	
	At3g18840.2	3	Translation	2037	23	6	0	6	6	0	0	2	9	2	2	1	0	1	2	0	
	At3g44790.1	3	Translation	975	0	0	0	6	1	2	0	0	2	1	0	0	0	1	0	2	
	At3g44805.1	3	Translation	747	0	0	0	0	0	0	0	0	0	0	0	0	0	0	0	0	
	At3g47050.1	3	Translation	1928	0	0	0	0	0	3	0	0	0	1	0	0	0	0	0	3	
	At3g47050.2	3	Translation	1953	0	0	0	0	0	3	0	0	0	1	0	0	0	0	0	3	
	At3g47070.1	3	Translation	669	351	107	127	180	191	47	111	105	191	114	25	23	14	17	54	47	
	At3g51325.1	3	Translation	704	10	2	7	9	10	0	1	1	7	3	1	0	1	1	3	0	
	At3g63460.1	3	Translation	3783	4990	1527	2802	3248	757	267	734	493	3142	563	360	330	310	305	214	267	[illegible]
	At3g63460.2	3	Translation	3777	4972	1526	2795	3213	754	265	730	490	3127	560	359	330	309	302	213	265	[illegible]
	At3g63460.3	3	Translation	3785	4954	1511	2773	3242	748	264	724	481	3120	554	358	327	306	305	211	264	[illegible]
	At4g15240.1	3	Translation	1815	94	55	99	80	19	8	14	13	82	14	7	12	11	8	5	8	
	At4g17200.1	3	Translation	1146	6	0	0	0	1	0	0	0	2	0	0	0	0	0	0	0	
	At4g37000.1	3	Translation	1068	685	197	424	322	112	19	66	58	407	64	49	43	47	30	32	19	
	At5g10180.1	3	Translation	2417	184	61	136	141	49	13	44	20	131	32	13	13	15	13	14	13	
*	At5g17910.1	3	Translation	4532	3195	1018	1311	1252	269	56	253	173	1694	188	231	220	145	118	76	56	
*	At5g17910.2	3	Translation	4757	3229	1022	1333	1273	269	56	257	174	1714	189	233	221	147	120	76	56	
	At5g39210.1	3	Translation	686	27	4	23	20	9	3	5	7	19	6	2	1	3	2	3	3	
**	At5g41960.1	3	Translation	874	92	35	87	113	11	2	9	12	82	9	7	8	10	11	3	2	

	At5g55520.1		3	Translation	2628	404	162	240	242	64	16	48	35	262	41	29	35	27	23	18	16
	At5g55520.2		3	Translation	2616	405	166	240	242	64	16	48	35	263	41	29	36	27	23	18	16
	At5g56200.1		3	Translation	1482	8	4	2	10	0	0	3	0	6	1	1	1	0	1	0	0
*	At5g57790.1		3	Translation	1407	560	190	178	178	51	11	31	22	277	29	40	41	20	17	14	11
*	At5g57790.2		3	Translation	916	507	174	169	148	50	8	24	18	250	25	37	38	19	14	14	8
	At5g58960.1		3	Translation	1880	1545	518	589	630	245	115	345	137	821	211	112	112	65	59	69	115
	At5g58960.2		3	Translation	1899	1480	499	573	583	246	115	340	137	784	210	107	108	63	55	69	115
	At5g58960.3		3	Translation	1823	1417	486	526	577	244	115	336	135	752	208	102	105	58	54	69	115
*	At5g01950.1		2	Cleavage	3387	872	246	487	463	137	21	82	57	517	74	63	53	54	44	39	21
	At2g07120.1		2,5	Cleavage	1062	0	0	0	0	0	0	0	0	0	0	0	0	0	0	0	0
	At3g18340.1	miR846	2,5	Cleavage	1086	0	0	26	0	0	0	0	0	7	0	0	0	3	0	0	0
	At3g44130.1		2,5	Cleavage	942	5	0	7	0	0	0	0	0	3	0	0	0	1	0	0	0
	At5g63020.1		1,5	Translation	2856	198	69	207	210	49	8	27	26	171	28	14	15	23	20	14	8

* padj < 0.05

** pvalue < 0.05

Supplementary Table 3. Location of circRNAs identified in at least three methods

circRNA	Chromosome	Start position	End position	Sense
At1g02560	1	539255	539479	+
At1g12080	1	4084568	4084663	+
At1g31810	1	11401293	11401343	-
At1g52360	1	19505448	19505672	+
At2g02410	2	633209	633280	+
At2g35940	2	15090857	15091786	-
At2g42170	2	17578221	17580061	+
At5g16880	5	5550910	5550959	+
At5g56950	5	23034400	23034518	+
At3g01800	3	287145	287213	+
At3g13990	3	4628775	4629836	-
At5g27720	5	9816179	9817163	+
